# The doctor vote: Interactions between political ideological preferences and healthcare reform strategies among U.S. physicians

**DOI:** 10.1016/j.hpopen.2024.100123

**Published:** 2024-07-20

**Authors:** Maitri Patel, Genevieve Lyons, Kara Fitzgibbon, B. Cameron Webb

**Affiliations:** a University of Virginia School of Medicine, 200 Jeanette Lancaster Way, Charlottesville, VA 22903, United States; b University of Virginia Weldon Cooper Center for Public Service, 2400 Old Ivy Road, Charlottesville, VA 22903, United States

**Keywords:** Physicians, Healthcare reform, Health policy, Survey

## Abstract

**Intro:**

Improving the American healthcare system has consistently predominated the domestic policy agenda in the United States for decades. However, physicians have traditionally played a small role in the U.S. legislative process despite their direct observations of the obstacles that cost, access, and quality can have on their patients and their care. The goal of this study was to examine the relationship between physician political ideological preferences and health policy reform options.

**Methods:**

We conducted a cross-sectional survey of 3,001 currently practicing U.S. physicians to predict how self-identification as liberal, moderate, or conservative impacted a physician’s policy preferences under the domains of cost, access, and quality.

**Results:**

A total of 536 (18.8%) out of 3,001 physicians responded to the survey. Overall, 32% of physicians identified as liberal, 43% as moderate, and 22% as conservative.

**Conclusion:**

Liberal-identifying physicians favored traditionally liberal reform ideas (a national health plan or public option), while conservative physicians preferred conservative policies (free market optimization). However, variation within political groups and domains of healthcare suggest that no single reform policy will be unanimously supported by every physician within a political group. Nonetheless, physicians are unanimously dissatisfied with the state of our current system, and physician-supported healthcare reform should be a national priority.

## Highlights

• Effective healthcare delivery is dependent on cost, quality, and access to care.
• Physicians are frontline observers of cracks in the healthcare system.
• Policy reforms include a nationalized plan, public option, and free market reform.
• Political ideology contributes to physician policy reform preference.
• Variation of reform preference among cost, quality, and access suggests further nuance.

## 1 Introduction

While improving the American healthcare system has remained atop the domestic policy agenda for much of the past three decades, there is a unique cache to health policy discussions in the current national landscape. Health reform debates in 2021 are taking place against the backdrop of a global pandemic that has accentuated challenges in public health infrastructure, healthcare access, provider reimbursement and health-related social factors. Additionally, these discussions flow in the wake of the contentious 2020 U.S. presidential election cycle, featuring passionate appeals for everything, from the repeal of the Affordable Care Act (ACA) to the enactment of a single payer healthcare system. In short, the only broad consensus is that the stakes are high.

As of 2019, the United States spends about $3.8 trillion on healthcare annually, 17.7 % of the national GDP [1]. Although the federal government spends almost twice as much on healthcare than the average OECD country [2], [3], a significant portion of economic burden still falls on individual patients. In 2018, the typical household spent $4,994.25 on healthcare—an increase of 101 % from just three decades ago [4]. While the uninsured rate decreased from 15.6 % to 8.5 % in the first eight years after the passage of the ACA, 29 % of insured adults are still considered “underinsured,” signifying their high deductibles and out-of-pocket medical expenses relative to their income were likely to result in significant financial burden or skipping care [5], [6]. Beyond the economic factors that influence an individual’s access to care, the quality of healthcare provided can be an additional obstacle that varies within different social, economic, and geographic groups [7], [8].

The current political landscape suggests 4 main health insurance models which can be adopted within the U.S. healthcare system: a nationalized health insurance program (e.g. Medicare-for-All), a public insurance option (e.g. the public option), improving free market competition through general price transparency, or continuing with the existing managed competition model with private insurances being the primary form of coverage. The nationalized health insurance model, which is currently reflected domestically by the U.S. Veterans Affairs and internationally by nations such as the United Kingdom, would allow public ownership of the healthcare arena. Patients would pay regulated insurance premiums to the federal government to receive healthcare coverage. The main strength of this model is universal healthcare coverage, while demerits include increased health service rationing and insufficient resources [9].

Creating a public option would mimic some of the effects created by the Affordable Care Act signed in 2010 by President Obama. The Affordable Care Act provides a universally accessible insurance plan intended to be competitive with private insurance plans. While the ACA was successful in decreasing the number of uninsured and underinsured individuals from 44.4 million (16.8 %) in 2013 to a historic low of 26.7 million (10.0 %) in 2016, there were also drawbacks [10]. The ACA allowed the government to deem certain insurance plans insufficient due to an inability to meet specified “essential health benefits” causing an increase in insurance premiums and destabilization of insurance coverage for almost three million individuals [11].

The goal of increasing price transparency is to provide patients with accurate estimates of the cost of care, thus allowing patients to “shop” for care, increasing competition, and driving down prices. The evidence of this effect is still unproven due to the often-urgent nature of healthcare delivery. This policy has been suggested to be especially successful in large urban settings in which multiple, competing health systems exist within a small radius of consumers [12].

Driving much of the action in this complex, challenging, and capable system is its primary operators: healthcare providers. These front-line providers directly observe the obstacles that cost, access, and quality can have on their patients, providing them a unique lens through which to evaluate the present—and advocate for the future—of the American healthcare system. While previous surveys have assessed physician ideological preferences or positions on the ACA [13], no study thus far has conducted a comprehensive investigation that integrates their ideological preferences with their health policy positions, particularly within the unique and dynamic context of today’s society and the global pandemic. The goal of this study is help inform federal efforts to reform the American healthcare system by aggregating the views and voices of the system’s physicians.

## 2 Methods

The Physician Engagement of Health Policy Issues Assessment (PEHPIA) survey was developed by the University of Virginia School of Medicine’s Health Equity, Law, and Policy Research (HELPR) laboratory in consultation with the University’s Center for Survey Research (CSR). The goal of this cross-sectional study was to assess the health policy attitudes and awareness of physicians practicing across the United States.

*Survey development and questionnaire design*

To frame the inquiry, a panel of national health policy experts were surveyed to submit their impressions of the most pertinent healthcare reform policy issues under the domains of cost, access, and quality. Responses consistently centered on overall healthcare costs, Medicare/Medicaid, prescription drug prices, payment methodologies, and the cost of health insurance. A survey tool was developed to explore the range of awareness and attitudes on these issues. The tool was focus-group tested among a small group of physicians at the University of Virginia. The questionnaire was administered in English only. The final questionnaire is included in the supplemental material.

*Survey Response*

Contact information for a probability-based sample of 3,001 physicians was purchased from the American Medical Association (AMA) Physician Masterfile, a database representative of all U.S.-accredited physicians. An advance letter was mailed on August 5, 2020, and the questionnaire packet (containing a cover letter, survey questionnaire, $2 unconditional incentive, and prepaid return envelope) was on August 12, 2020. A reminder postcard was sent to non-respondents on August 25, 2020, followed by a second packet to non-respondents on September 3, 2020. This packet contained the survey questionnaire, postage-paid return envelope, and a modified cover letter that included a short URL and unique access code to allow participants to complete the survey online (via Qualtrics). As a final mode of contact, telephone calling was conducted from September 9 to September 23, 2020 to a subset of non-respondents. Completed questionnaires were collected and entered in the database until November 28, 2020.

CSR performed data entry and validation of returned paper questionnaires. All data preparation was conducted in SPSS.

*Statistical analysis*

Final data downloaded from the database on November 27, 2020, were analyzed using SAS, version 9.4 (SAS Institute Inc) and R version 4.0.4. The survey used a 5-point scale to measure political ideological preferences (very liberal, liberal, moderate, conservative, and very conservative). Very liberal and liberal as well as very conservative and conservative were combined in final analyses to create three categorizations for ideological preference.

Descriptive prevalence analyses of reform method distribution among liberals, moderates, and conservatives were calculated. Chi-square tests measuring the association between ideological preferences and reform methods were conducted and odds ratios were estimated for each political reform response method. Additionally, a 5-point Likert scale was used to assess levels of support for a national health plan, public option, ACA revision, and free market optimization.

Table 1 presents the counts of final AAPOR dispositions of all physicians in the sample. Physicians were excluded from the study if they were not currently practicing at the time of survey administration.

**Table 1:** **Final Dispositions.** Shown are final counts and percentages of all physicians contacted for participation in this survey.

AAPOR Code	Eligibility	Final Disposition	Count	Percent
1.1	Eligible	Complete	536	17.9 %
2.11	Eligible	Refusal	48	1.6 %
2.3	Eligible	Other, non-refusal	929	31.0 %
3.25	Unknown Eligibility	Bad address, cannot be delivered	82	2.7 %
3.19	Unknown Eligibility	Non-interview, nothing returned	1330	44.3 %
4.1	Not Eligible	Ineligible	76	2.5 %
Total			3001	100 %

## 3 Results

Among the 3,001 physicians who were mailed the PEHPIA survey, 536 successfully completed and returned the survey from 44 different states. Following the American Association for Public Opinion Research (AAPOR) disposition codes and response rate calculations, this number of completed surveys yields an estimated response rate of 18.8 %. The margin of error for the sample is approximately +/- 4.2 percent at the 95 percent level of confidence. Demographics of respondents are summarized in Table 2. The median age was 55 (range, 30 to 87) with 376 respondents identifying as male (70.15 %) and 150 respondents identifying as female (27.99 %). Regarding political ideological preference, 174 respondents (32.46 %) selected liberal, 230 (42.91 %) chose moderate, and 119 (22.2 %) identified as conservative. The majority of participants had been practicing for over 20 years (304 [56.72 %]), practiced within a specialty (316 [58.96 %]), had some patients experiencing economic barriers in the year leading up to the COVID-19 pandemic (382 [71.27 %]), and had high continuity of care (305 [56.90 %]). Physicians were evenly split between a fee-for-service (41.98 %) and fixed salary (43.47 %) payment model. Nearly all physicians surveyed reported at least some level of interest in US healthcare policy (520 [97.01 %]; Table 2).

**Table 2:** **Respondent Characteristics.** Shown are demographic characteristics of the respondents, including age, gender, sex, political ideological preference, specialty, years practicing, fee model, and interest in U.S. health policy.

Characteristic	Median	Minimum	Maximum
Age	55	30	87
			
**Characteristic**	**Item**	**Frequency**	**Percentage**
Gender	Missing/refused	8	1.49
	Male	376	70.15
	Female	150	27.99
	Other	2	0.37

Hispanic, Latino, or Spanish Origin	Missing/refused	13	2.43
	Yes	35	6.53
	No	488	91.04

Race*	Asian or Asian American	85	
	Black or African American	21	
	Native American or American Indian	4	
	Native Hawaiian or Pacific Islander	2	
	White	386	
	More than one race	11	
* Participants Were Permitted to Indicate More Than One Race, So Percentages Were Not Provided. The Race Totals May Add Up to More Than the Total Sample Size.			

Political Ideological Preference	Missing/refused	13	2.43
	Very Liberal	21	3.92
	Liberal	153	28.54
	Moderate	230	42.91
	Conservative	96	17.91
	Very Conservative	23	4.29

Years Practicing Medicine	Missing/refused	3	0.58
	Currently a Resident	1	0.19
	0–5 years	42	7.84
	6–10 years	45	8.40
	11–15 years	71	13.25
	16–20 years	70	13.06
	> 20 years	304	56.72

Specialty	Missing/refused	5	0.93
	Primary Care / Frontline	187	34.89
	Specialty / Subspecialty	316	58.96
	Indirect patient care specialty	28	5.22

Patients With Financial Barriers in The Year Leading Up to Covid Pandemic	Missing/refused	5	0.93
	No patients	34	6.34
	Some patients	382	71.27
	Most patients	70	13.06
	All patients	3	0.56
	I have not seen patients in the last year	2	0.37
	Cannot estimate	40	7.46

Continuity Of Care	Missing/refused	6	1.12
	No direct patient contact	22	4.10
	No continuity	70	13.06
	Some continuity	133	24.81
	High continuity	305	56.90

Preventative Care Negatively Impacted	Missing/refused	6	1.12
	Not at all	15	2.80
	Some extent	133	24.81
	Moderate extent	149	27.80
	Significant extent	136	25.37
	Not applicable to my practice	97	18.10

Current Payment Method	Missing/refused	10	1.87
	Fee-for-service	225	41.98
	Capitation	15	2.80
	Fixed salary	233	43.47
	Pay-for-performance	53	9.89

Interest in U.S. Healthcare Policy	Missing/refused	4	0.75
	Not interested	12	2.24
	Somewhat interested	126	23.51
	Moderately interested	189	35.26
	Very interested	205	38.25

Results of a chi-square test indicated a statistically significant association between political ideological preferences and preferred strategies for health policy reform under all three domains—access (p < 0.0001), cost (p < 0.0001), and quality (p < 0.0001), which are all far below a Bonferroni-adjusted significance level of 0.0167. We found that conservatives were significantly less likely to support a national health plan or a public option when compared to non-conservative physicians under both the access and quality domains. However, under the domain of cost, the only strategy for health policy reform with a statistically significant difference between conservative and non-conservative physicians was the likelihood of supporting a national health plan.

The odds ratio estimates indicate how much more or less likely an ideological group was to support a particular reform option, compared to other groups. Under the domain of access, conservative physicians were statistically less likely than liberals to support a national health plan (OR = 0.04 or 96 % reduction in odds, 95 % CI 0.01 – 0.18) or a public option (OR 0.18 or 82 % reduction in odds, 95 % CI 0.05 – 0.72). Conservatives were statistically less likely than moderates to support a national health plan (OR = 0.08 or 92 % reduction in odds, 95 % CI 0.05–0.72) or a public option (OR 0.22 or 78 % reduction in odds, 95 % CI 0.07 to 0.71). Liberals and moderates did not significantly differ in their odds of preferring particular reform strategies.

Under the domain of cost, conservative physicians were statistically less likely than liberals to prefer the national health plan (OR = 0.06 or 94 % reduction in odds, 95 % CI 0.01 to 0.34), but their odds of preferring other reform options were not significantly different. Compared to moderates, they were statistically less likely to prefer a national health plan (OR 0.11 or 89 % reduction in odds, 95 % CI 0.02 to 0.56) but did not significantly differ in their odds of preferring other reform strategies.

Under the domain of quality, conservative physicians were statistically less likely than liberal physicians to support a national health plan (OR = 0.06 or 94 % reduction in odds, 95 % CI 0.02 – 0.19) or a public option (OR 0.19 or 81 % reduction in odds, 95 % CI 0.07 % − 0.56). Compared to moderates, they were statistically less likely to support a national health plan (OR 0.12 or 78 % reduction in odds, 95 % CI 0.4 to 0.38), and statistically less likely to support a public option (OR 0.29 or 71 % reduction in odds, 95 % CI 0.12 to 0.75). Liberals and moderates did not significantly differ in their odds of a particular reform strategy preference (Table 3).

**Table 3:** **Odds Ratios comparing Political Ideology and Policy Preference.** Shown are calculated odds ratios for each combination of comparison between different political ideological preferences according to their health policy reform method of choice under our three health domains. Bolded values indicate statistical significance.

			Estimate	95 % Confidence Limits	
**Access**	Conservative vs Liberal	***National Health Plan***	***0.04***	***0.01***	***0.18***
		***Public Option***	***0.18***	***0.045***	***0.72***
		Free Market Optimization	2.01	0.46	8.79
	Conservative vs Moderate	***National Health Plan:***	***0.08***	***0.02***	***0.30***
		***Public Option***	***0.22***	***0.07***	***0.71***
		Free Market Optimization	0.80	0.25	2.61
	Liberal vs Moderate	National Health Plan	2.00	0.46	8.64
		Public Option	1.23	0.28	5.25
		Free Market Optimization	0.40	0.08	1.93

**Cost**	Conservative vs Liberal	***National Health Plan***	***0.06***	***0.01***	***0.34***
		Public Option	0.34	0.06	1.95
		Free Market Optimization	2.03	0.34	12.16
	Conservative vs Moderate	***National Health Plan***	***0.11***	***0.02***	***0.57***
		Public Option	0.29	0.06	1.37
		Free Market Optimization	0.95	0.20	4.46
	Liberal vs Moderate	National Health Plan	2.01	0.33	12.29
		Public Option	0.86	0.14	5.35
		Free Market Optimization	0.47	0.07	3.08

**Quality**	Conservative vs Liberal	***National Health Plan***	***0.06***	***0.02***	***0.19***
		***Public Option***	***0.20***	***0.07***	***0.56***
		Free Market Optimization	2.33	0.80	6.82
	Conservative vs Moderate	***National Health Plan***	***0.12***	***0.04***	***0.38***
		***Public Option***	***0.29***	***0.12***	***0.75***
		Free Market Optimization	1.27	0.54	3.00
	Liberal vs Moderate	National Health Plan	2.25	0.85	6.00
		Public Option	1.54	0.58	4.11
		Free Market Optimization	0.54	0.19	1.58

**Fig. 1:**
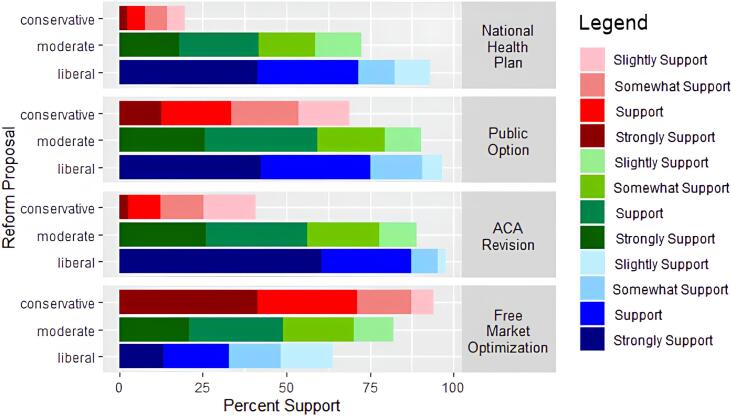
**Levels of Physician Support for Various Current Health Policy Reform Options by Political Ideological Preference.** Shown are stacked support levels of physicians regarding a national health plan, e.g. Medicare-for-All, a government-administered health insurance option that would compete with private insurance in the free market (a public option), the revision of the Affordable Care Act, and efforts to optimize the ability of free market forces to drive down the cost of health coverage and care (including greater price transparency, market deregulation and the elimination of mandates for coverage).

In the assessment of preferred health reform approach, 410 (78.7 %) physicians at least somewhat supported efforts focused on optimizing free market forces (111 [63.8 %] liberal; 188 [82.1 %] moderate; 111 [94.1 %] conservative). Most physicians (419 [81.2 %]) supported the revision of the Affordable Care Act (168 [97.7 %] liberal; 204 [89.1 %] moderate; 47 [40.9 %] conservative). Additionally, 458 (87.6 %) physicians at least somewhat supported a public option (168 [96.6 %] liberal; 208 [90.4 %] moderate; 82 [68.9 %] conservative). Many physicians (340 [66.7 %]) also favored a national health plan (160 [93.1 %] liberal; 157 [71.4 %] moderate; 23 [19.5 %] conservative; Fig. 1).

**Fig. 2:**
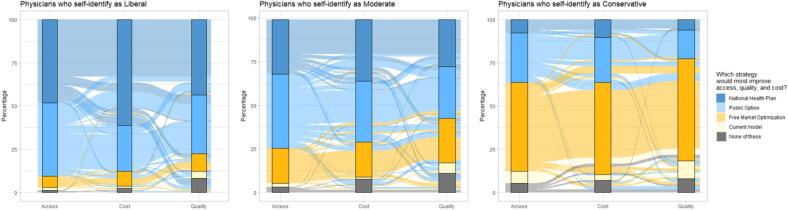
**Physician Preference of Healthcare Reform by Political Ideological Preference.** Shown are three panels (by ideological preference) displaying which healthcare reform option physicians most closely aligned with when given the choice between a national health plan (e.g., Medicare-for-All), a widely available public health insurance option (a public option), free market optimization through general price transparency, managed competition model with private insurances being the primary form of coverage (our current model), or none of the above.

Across all three domains of healthcare—access, cost, and quality—liberals, moderates, and conservatives consistently maintained their preferred option (Fig. 2). The liberal top choice for access-focused healthcare reform was a national health plan (85 [48.85 %]) followed closely by a public option (73 [41.95 %]). Liberals favored a national health plan for both a cost (107 [61.49 %]) and quality-focused health reform approach (74 [43.53 %]). Moderates favored a public option in both the access (100 [43.67 %]) and quality domains (70 [30.84 %]) but were tied between a national health plan and public option under a cost-based reform strategy (80 [35.09 %] for both). The top choice for conservatives across all three domains was a focus on free market optimization (59 [50.0 %] for access; 61 [52.14 %] for cost; 68 [58.62 %] for quality; Fig. 2).

## 4 Discussion

The results of this survey indicated that physicians across America represent a range of political ideologies and express a wide variety of views on the correct course for the future of the nation’s healthcare system. Results of the chi-square test and estimated odds ratios indicated a strong association between political ideology and preference for specific approaches to healthcare reform. However, they fail to recognize the larger picture—most physicians do support reform and do not believe that our current system is best.

Fig. 2 visually demonstrates that despite overall consistency between ideological preferences and policy reform, there exists variation in preference for reform policy proposals, both within groups of ideological preferences and across different domains of healthcare. This variation indicates that although political ideological preferences are a strong predictor, there are other factors that impact a physician’s decision to support a specific reform option. The fact that these views vary across cost, access, and quality speaks to the belief that different reforms would have varying degrees of impact on key issues in healthcare.

Based on a holistic interpretation of our estimated odds ratios, only a few ideological differences are statistically significant. The preferences of liberals and moderates are not statistically different in any domain. Conservatives have significant disagreement with moderates and liberals but only with respect to a national health plan (uniformly) or a public option (in terms of access and quality, but not cost). The implication is that some conservative physicians believe that a public option could be viable in terms of reducing cost. Generally, physicians appear to have experience-based—not merely ideological—ideas about which reforms could most effectively alleviate the shortcomings of the current American healthcare system.

Health reform studies in years past have typically supported the stratified variation in physician reform policy preferences and the sense of overall dissatisfaction with the current system represented by our findings. Previous studies examining the role of political lean among physicians and their corresponding views on the ACA, typically regarded as a liberal policy reform option, found a similar predilection for favor among liberal physicians. [13] In addition, when polled regarding their views on a nationalized plan and a public option, medical students who self-segregated into political affiliations showed similar results, including not only overall predisposition based on political lean, but also variation in reform support that was independent of their lean preference. [14] These studies along with ours demonstrate that health policy reform is a imminent and necessary change that is dependent on many characteristics that define a physician, which includes but is not limited to political lean.

Physicians are not traditionally involved in the legislative side of politics. Approximately 25 % of physicians are involved in policy at either a local, state, or national level, and currently in the 117th Congress, only 13 representatives and four senators are physician-represented out of 435 congressmen. [13], [15] By and large, the majority of individuals deemed responsible for changing healthcare policies in the U.S. are not healthcare workers themselves, and have not personally experienced the profound impact healthcare has on individual patients.

Limitations

Almost all respondents (97 %) reported at least a mild interest in healthcare policy, suggesting that our results may under-represent physicians who are not interested in healthcare policy. Additionally, we recognize that our sample was demographically skewed, representing a population of physicians who was primarily Caucasian, male, and had been in practice for more than 20 years; however, our sample generally aligned with the current population of practicing physicians with respect to race (56.2 % White), gender (64 % male), and age (mean age of 51.7) [16], [17].

Conclusion

Overall, this study highlights the reality that no single plan will be unanimously supported by all physicians; many liberal physicians support free market optimization, and some conservative physicians support a national health plan. Complex interactions of various identities and insights mold a physician’s views on health policy, thus requiring greater nuance to inform future reform discussions and meaningful progress. Overall, we observed that doctors are open to many policy reforms, so long as it involves changing our current system.

With a dearth of studies directly examining the impact of political ideological preferences on one’s preferred health policy reform strategy, this analysis not only adds to the current understanding, but highlights the need for further research. One such opportunity would be examining how other demographic factors are related to physician reform preference. Future studies could also consider dynamics of physician practice, such as payment model, financial concerns among primary patient population, and type of practice (academic, private, out-patient, in-patient).

## Declaration of competing interest

The authors declare that they have no known competing financial interests or personal relationships that could have appeared to influence the work reported in this paper.
