# Supplementary material for: The doctor vote: Interactions between political ideological preferences and healthcare reform strategies among U.S. physicians
Source: Health Policy Open. 2024 Jul 20;7:100123. doi: 10.1016/j.hpopen.2024.100123 (PMC11325352; doi:10.1016/j.hpopen.2024.100123)
Supplement: Supplementary Data 1 [file mmc1.pdf]

Physician Engagement of Health Policy Issues Survey

American health care is changing. Medical practice, payment, and policies have undergone some fundamental transformations in recent years. As a healthcare provider, you sit front and center for the very real impacts of all of this change. Thank you for taking the time to share your perspective and expertise on American health policy by completing this questionnaire. We appreciate your time.

The survey's purpose: Understand physicians' attitudes toward, and engagement with, various U.S. health policy issues.

General instructions:

- Circle the number of your response to the question or check the boxes that apply.
- The survey should take about 15 minutes to complete.
- Your responses are completely confidential and will never be identified with you individually.
- You can decline to take part in the survey or skip any questions you do not wish to answer. Participation in this survey will have no effect on your employment.
- When you finish answering the questions, put your survey in the pre-paid envelope provided and drop it in any mailbox. **No postage is required.**
- Withdrawal is not permitted after the submission of the questionnaire.
- If you have questions, you can contact the Center for Survey Research: surveys3@virginia.edu, 434-243-5232.

Thank you for your time and consideration. Your participation has the potential to impact the national health policy discourse!

Dr. B. Cameron Webb
Department of Public Health Sciences
University of Virginia School of Medicine
Telephone: (434)924-1938
bcw8q@hscmail.mcc.virginia.edu

Your participation in this survey is voluntary. There are no known risks or direct benefits associated with your participation. However, your individual cooperation is very important to the success of this study and is greatly appreciated! We thank you for your time.

This survey has been approved by the University of Virginia's Institutional Review Board for the Social and Behavioral Sciences (Project #3546). If you have questions about this study, contact: Tonya R. Moon, Ph.D. (Chair, IRB-SBS), 434-924-5999, irbsbshelp@virginia.edu.

A. Professional Background

To begin, please answer the following questions about your medical experience.

A1. Years practicing medicine (since finishing residency):

1 Currently a Resident
2 0 - 5 years
3 6 - 10 years
4 11 - 15 years
5 16 - 20 years
6 > 20 years

A2. What specialty of medicine do you currently practice?

1 Primary Care Physician/Frontline Provider (e.g. Hospitalist, Family Practice, Internal Medicine, General Pediatrics)
2 Specialty/Subspecialty (e.g. Surgery, Psychiatry, Emergency, OB/GYN, etc.)
3 Indirect Patient Care Specialty (e.g. Radiology, Pathology, etc.)
4 Do not currently practice

A2a. (**If not currently practicing**) We appreciate your interest in the survey, but we are only surveying actively practicing physicians at this time. Please stop here and return the survey in the enclosed envelope. Thank you for your time.

A3. Please write out the state in which you currently practice.

____

A4. In the year leading up to the Covid-19 pandemic, how many of your patients do you estimate faced out-of-pocket healthcare costs that were a significant barrier to their care? Here, "barrier" is defined as the patient undergoing significant financial hardship to receive care.

1 No patients
2 Some patients
3 Most patients
4 All patients
5 I have not seen patients in the last year
6 Unable to estimate

A5. On average, how would you describe the continuity of your patient relationships prior to Covid-19?

1 No direct patient contact
2 No continuity (see patient once)
3 Some continuity (see patient a few times)
4 High continuity (see patient regularly)

A6. Overall, to what extent has preventative care (e.g., routine visits, vaccinations, screenings, etc.) for your patients been negatively impacted by the Covid-19 pandemic?

1 Not at all
2 Some extent
3 Moderate extent
4 Significant extent
5 Not applicable to my practice

A7. To the best of your ability, select the option that best reflects your current payment method.

1 Fee-for-service
2 Capitation
3 Fixed salary
4 Pay-for-performance

A8. A set of six influential values, listed below, have shaped the United States healthcare system for decades. Which of these values do you consider the most important in the future of our healthcare system? Select your top **two**.

- ❑ Professional autonomy
- ❑ Patient autonomy
- ❑ Consumer sovereignty
- ❑ Patient advocacy
- ❑ High-quality care
- ❑ Access to care

A9. Overall, how would you rate your interest in U.S. healthcare policy?

1 Not interested
2 Somewhat interested
3 Moderately interested
4 Very interested

A10. How has your interest in U.S. healthcare policy changed, if at all, since the start of the Covid-19 pandemic?

1 Significantly increased
2 Increased
3 No change
4 Decreased
5 Significantly decreased

A11. Overall, how would you rate your knowledge of U.S. healthcare policy?

1 Not at all knowledgeable
2 Somewhat knowledgeable
3 Moderately knowledgeable
4 Very knowledgeable

B. Health Policy Attitudes

Over the past few years, there have been many changes in U.S. healthcare policy. Please choose the option that best aligns with your view regarding the following questions.

B1. To what extent do you agree that Medicaid work requirements should be mandated in all states?

1 Disagree
2 Somewhat disagree
3 Neutral
4 Somewhat agree
5 Agree

B2. To what extent do you agree that the federal government should pass legislation to limit individuals' out-of-pocket costs related to health coverage, including insurance premiums, co-pays and deductibles?

1 Disagree
2 Somewhat disagree
3 Neutral
4 Somewhat agree
5 Agree

B3. To what extent do you agree that encouraging health insurance as an employee benefit (i.e., employer-sponsored insurance) is the best way to ensure health coverage for non-elderly adults in the United States?

1 Disagree
2 Somewhat disagree
3 Neutral
4 Somewhat agree
5 Agree

B4. To what extent do you agree that allowing the federal government to negotiate the price of prescription drugs is a key component of ensuring lower drug costs for Americans?

1 Disagree
2 Somewhat disagree
3 Neutral
4 Somewhat agree
5 Agree

B5. To what extent do you support a national health plan where all Americans would receive medical coverage from a single government plan, e.g. Medicare-for-All?

1 Do not support
2 Slightly support
3 Somewhat support
4 Support
5 Strongly support

B6. To what extent do you support a government-administered health insurance option available to all Americans that would compete with private insurance in the free market (e.g. a public option)?

1 Do not support
2 Slightly support
3 Somewhat support
4 Support
5 Strongly support

B7. To what extent do you support the revision of the Affordable Care Act to ensure that all of the major provisions of the 2010 law are appropriately funded and enforced?

1 Do not support
2 Slightly support
3 Somewhat support
4 Support
5 Strongly support

B8. To what extent do you support robust efforts to improve the ability of free market forces to drive down the cost of health coverage and care (including greater price transparency, market deregulation and the elimination of mandates for coverage)?

1 Do not support
2 Slightly support
3 Somewhat support
4 Support
5 Strongly support

B9. Which of the strategies listed below, if any, would you support in an effort to lower drug prices? (Select a single stance for each strategy).

	**Support**	**Do Not Support**	Not Sure
**a. Absolute price ceilings**	***1***	***2***	*9*
**b. Expedited reviews and inspections of generic drugs**	***1***	***2***	*9*
**c. Increased price transparency**	***1***	***2***	*9*
**d. Stricter FDA patent renewal policies**	***1***	***2***	*9*
**e. Reference pricing against other nations**	***1***	***2***	*9*
**f. Government-regulated cost-effectiveness analysis**	***1***	***2***	*9*

The following questions refer specifically to the approaches for providing health care coverage in the current political discourse. Please select the response that most closely captures your view.

B10. Which of the following methods would best increase access to quality healthcare for all Americans?

1 Nationalized health insurance program (e.g., Medicare-for-All)
2 A widely-available public health insurance option (e.g., the public option)
3 Free market competition through general price transparency
4 Managed competition model with private insurances being the primary form of coverage (our current model)
5 None of the above

B11. Which of the following methods would best lower direct costs to the patient, including but not limited to premiums, taxes, and out-of-pocket payment?

1 Nationalized health insurance program (e.g., Medicare-for-All)
2 A widely-available public health insurance option (e.g., the public option)
3 Free market competition through general price transparency
4 Managed competition model with private insurances being the primary form of coverage (our current model)
5 None of the above

B12. Which of the following methods would best allow you, as a physician, to provide the highest quality care for your patients?

1 Nationalized health insurance program (e.g., Medicare-for-All)
2 A widely-available public health insurance option (e.g., the public option)
3 Free market competition through general price transparency
4 Managed competition model with private insurances being the primary form of coverage (our current model)
5 None of the above

B13. If you selected a nationalized health insurance program in B10, B11, or B12, how do you think a single-payer system should be funded?

1 Employer-based Annual Premiums
2 Household-based Annual Premiums
3 Income taxes
4 General tax revenue
5 I did not select nationalized system

C. Health Policy Awareness

The following questions assess your awareness of U.S. healthcare policy as it stands today. Please answer the questions to the best of your ability. We ask that you not look up the information.

C1. As of October 2019, how many states have adopted *and* implemented the Medicaid expansion?

1 19 States
2 28 States
3 34 States
4 42 States

C2. In 2019, approximately how much of the nation's Gross Domestic Product (GDP) was spent on health-related expenses?

1 10%
2 20%
3 25%
4 30%

C3. In 2019, what percent of the U.S. population utilized employer-sponsored insurance?

1 38%
2 50%
3 67%
4 76%

C4. At present, waste accounts for approximately what percent of health expenditures?

1 25%
2 35%
3 45%
4 50%

C5. In 2019, how many individuals in the U.S. were uninsured?

1 10.2 million (3.1%)
2 27.5 million (8.8%)
3 36.5 million (11.3%)
4 45.6 million (14.1%)

C6. In 2018, what percent of the approximately 3.3 trillion dollars of healthcare expenditures was spent on prescription drugs?

1 10%
2 15%
3 20%
4 25%

C7. For Medicaid funding, what is the minimum Federal Medicaid Matching Rate (FMAP)?

1 25%
2 30%
3 40%
4 50%

C8. Match the parts of Medicare with their functions. Select only one part for each function.

	Part A	Part B	Part C	Part D
**a. Prescription Drugs**	1	2	3	4
**b. Physician Payments**	1	2	3	4
**c. Hospital Costs**	1	2	3	4
**d. Medicare Advantage**	1	2	3	4

C9. Who is eligible for Medicaid benefits?

1 Low-income families
2 Pregnant women
3 Patients with end-stage renal disease
4 Both A and B
5 All of the above

D. Closing Demographics

Thank you for your responses thus far. We have just a few final questions about you. Recall that all information provided will be kept strictly confidential.

D1. How old are you? ______________

D2. With which gender do you identify?

1 Man
2 Woman
3 I prefer to provide my own description:__________________________________

D3. Are you of Hispanic, Latino, or Spanish origin?

1 Yes
2 No

D4. With which race/ethnicity do you identify? Select all that apply.

- ❑ Asian or Asian American
- ❑ Black or African American
- ❑ Native American or American Indian
- ❑ Native Hawaiian or Pacific Islander
- ❑ White
- ❑ I prefer to provide my own description: ___________________________________

D5. How would you classify your political lean?

1 Very Liberal
2 Liberal
3 Moderate
4 Conservative
5 Very Conservative

D6. Which of the following sources do you use to gain knowledge on current U.S. healthcare policy? Select up to **two** options.

- ❑ Academic journals/books
- ❑ Citizen groups
- ❑ Professional/Academic events
- ❑ Professional colleagues
- ❑ Podcasts
- ❑ News media, digital or printed
- ❑ Radio
- ❑ Friends and Family
- ❑ Social media (Facebook, Twitter, Instagram, online blogs, etc.)
- ❑ Not applicable
- ❑ Other: ____

Thank you very much for taking the time to complete this survey.
Your responses are valued and very much appreciated!

No postage is required to mail back this questionnaire.

Please use the envelope provided.

Lost your envelope?

Please return the questionnaire to us at:
Center for Survey Research
University of Virginia
P.O. Box 400767
Charlottesville, VA 22904-4767

1st Packet: [CSRID]
